# Supplementary material for: Electrical and Optical Properties of CaTi1−yFeyO3−δ Perovskite Films as Interlayers for Optoelectronic Applications
Source: Materials (Basel). 2022 Sep 21;15(19):6533. doi: 10.3390/ma15196533 (PMC9570815; doi:10.3390/ma15196533)
Supplement: Supplementary file 1 [file materials-15-06533-s001.zip › materials-1865326-supplementary.pdf]

materials

MDPI

Supplementary Information

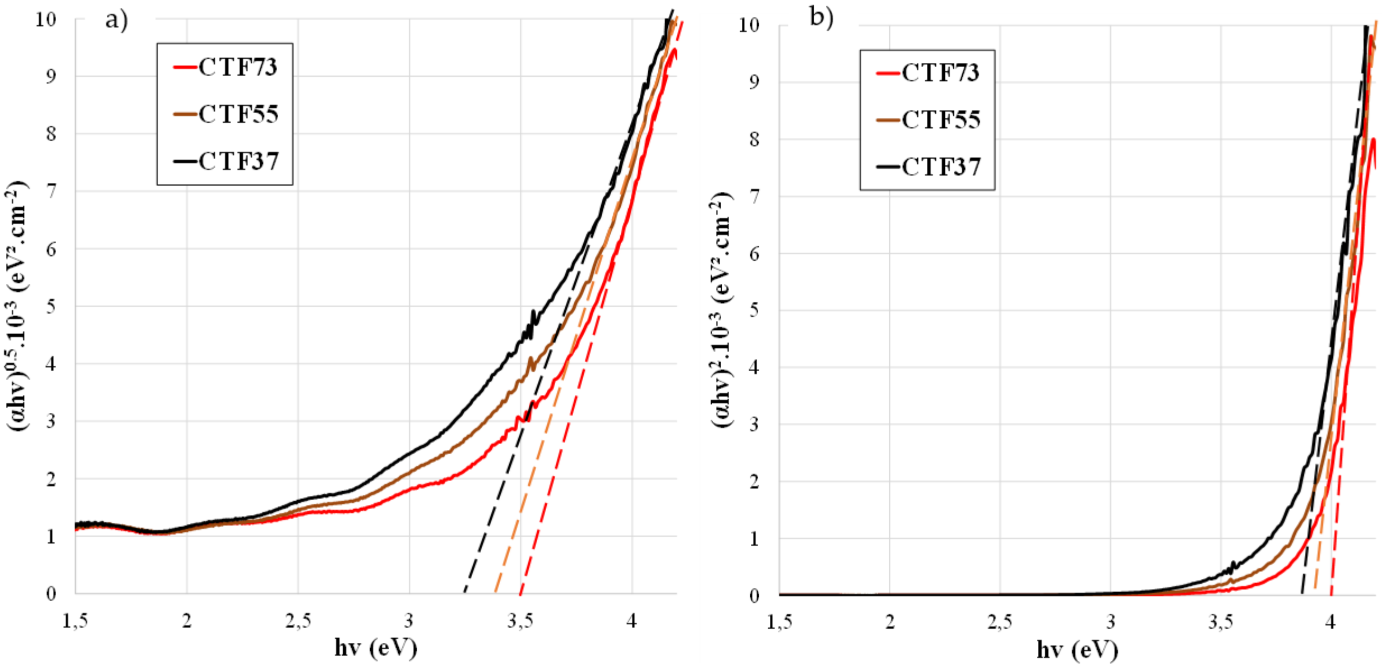

Figure S1. Tauc plots (a) with r=0.5, (b) with r=2, and associated fits for CTF73, CTF55, and CTF37.

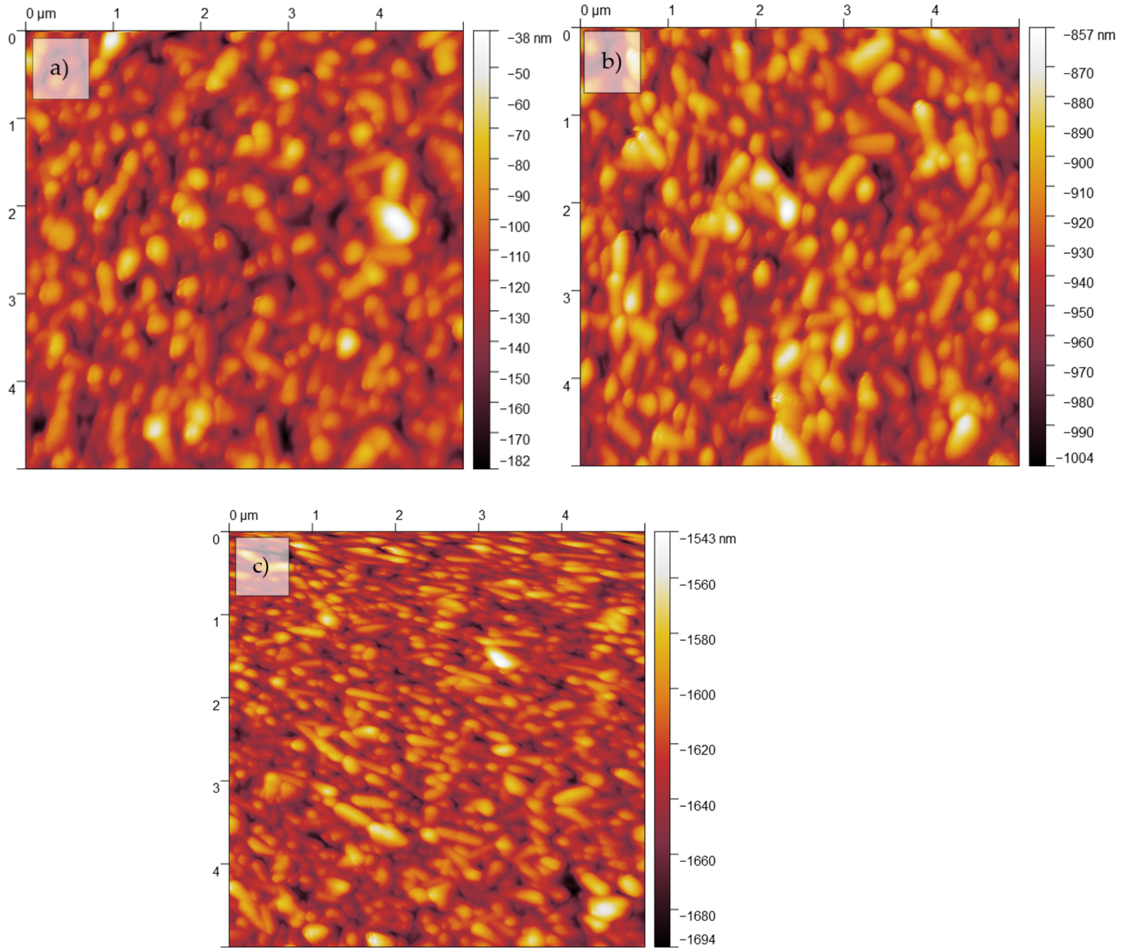

Figure S2. AFM topography of (a) CTF37, (b) CTF55, and (c) CTF37 on FTO.

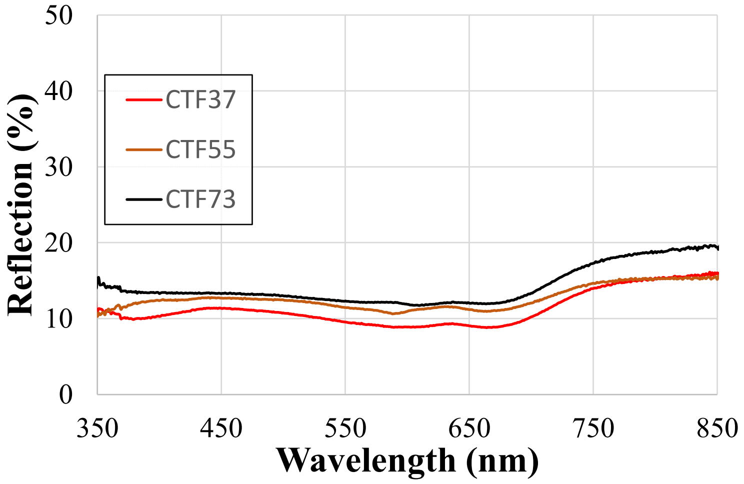

Figure S3. Reflection of CTF layers deposited on sapphire.
